# Dental Color-Matching Ability: Comparison between Visual Determination and Technology

**DOI:** 10.3390/dj12090284

**Published:** 2024-09-03

**Authors:** Maria Menini, Lorenzo Rivolta, Jordi Manauta, Massimo Nuvina, Zsolt M. Kovacs-Vajna, Paolo Pesce

**Affiliations:** 1 Department of Surgical and Diagnostic Sciences (DISC), University of Genoa, 16126 Genova, Italy; maria.menini@unige.it; 2 Private Practice, 16100 Genova, Italy; 4348863@studenti.unige.it (L.R.); jordi@styleitaliano.org (J.M.); 3 Private Practice, 10128 Turin, Italy; nuvina@tin.it; 4 Department of Information Engineering (DII), University of Brescia, Via Branze 38, 25123 Brescia, Italy

**Keywords:** color, tooth color, prothethic dentistry, esthetic

## Abstract

Background: The choice of the correct color is of paramount importance in esthetic dentistry; however, there is still no consensus on the best technique to determine it. The aim of the present study is to compare the accuracy of a recently introduced colorimeter in shade matching with human vision. In addition, possible variables affecting color-matching by human eye have been analysed. Methods: 18 disc-shaped composite samples with identical size and shape were produced from a composite flow system (Enamel plus HriHF, Micerium): Nine were considered control samples (UD 0-UD 6), and nine were test samples with identical flow composite shade to the control ones. Parallelly, 70 individuals (dental students and dental field professionals) were individually instructed to sit in a dark room illuminated with D55 light and to perform visual shade matching between control and test discs. An error matrix containing ΔE_94_ between control and test discs was generated, containing four match-clusters depending on perceptibility and acceptability thresholds. The frequency and severity of errors were examined. Results: The colorimeter achieved a 100% perfect matching, while individuals only achieved a 78%. A higher occurrence of mismatches was noted for intermediate composite shades without a statistically significant difference. No statistically significant differences were reported for age, sex, and experience. A statistically significant difference was present among the Optishade match and the visual determination. Conclusions: The instrumental shade-matching evaluation proved to be significantly more reliable than the human visual system. Further research is needed to determine whether the same outcomes are achieved in a clinical setting directly on patients.

## 1. Introduction

The color and appearance of a tooth is determined by the interplay of multiple optical properties. Human color perception relies on the subjective capacity of the visual system to merge and understand the way light interacts with objects. When light reflects and enters the observer’s eyes, its specific qualities elicit the retina, resulting in a subjective color perception [1]. Tooth color ensues from the absorption, transmission, and reflective properties of enamel and the subjacent dentine, plus the scattering volume of light [2], i.e., illuminating light follows highly irregular light paths through the tooth before it emerges at the surface of incidence and reaches the eye of the observer [3,4]. Odor et al. defined both enamel prisms and dentinal tubules as being comparable to optical fibers [5]. The mechanism by which different reflections and transmissions of light combine to form the colors seen in human teeth is a complex process that is not yet fully understood [6].

Based on the international standard color specification system by the CIE (Commission Internationale de l’Eclairage, that is, the International Commission on Illumination), tooth color benefits from color coordinates that are relatively easy to interpret, with the primary considerations regarding lightness (L*), red (+a*), and yellow (+b*) [7]. The dental color space can be visualized as an atypical, asymmetric bean shape in the complete L*a*b* color space. It displays limited chromaticity and is predominantly concentrated in the bright, yellow-red zone, with most of the space being near to the neutral axis [8].

The aesthetics of the teeth is of significant concern for numerous people, with many expressing apprehensions regarding the appearance and color of their dentition [9,10].

Researches demonstrated that a variety of adult demographics have shown 20–50% dissatisfaction with the overall mouth impression [11,12] and, specifically, 20–60% are uncomfortable with the color of their dentition [13,14,15]. Undoubtedly, the ultimate goal of tooth color measurement in modern aesthetic dentistry is to achieve a level of accuracy that allows a restoration to blend seamlessly with the patient’s natural dentition, without any detectable color discrepancies [16,17].

In 1994 the CIE proposed a new formula: the CIE_94_ (ΔE_94_) formula employs the LCh* color space to evaluate differences in color, specifically with regard to lightness, chroma, and hue, as computed from L*a*b* coordinates:

$$\Delta E_{94}^{*}=\sqrt{{\left(\frac{\Delta L^{*}}{K_{L}S_{L}}\right)}^{2}+{\left(\frac{\Delta C_{ab}^{*}}{K_{C}S_{C}}\right)}^{2}+{\left(\frac{\Delta H_{ab}^{*}}{K_{H}S_{H}}\right)}^{2}}$$

where

$$\Delta L^{*}=L^{*}-L_{ref}^{*}$$

$$C_{1}^{*}=\sqrt{{a_{1}^{*}}^{2}+{b_{1}^{*}}^{2}}$$

This formula was updated in 2000 with the new CIEDE2000.

The perceptibility threshold (PT) is a measure of the smallest color difference that can be detected by the human eye, with a 50:50 threshold indicating the color difference that can be perceived by 50% of observers, while the other 50% will not detect any difference in color. Achieving a color match that falls at or below the 50:50 perceptibility threshold is considered a key goal in dental restorations, as it indicates that the restoration blends perfectly with the surrounding teeth tissues.

Most studies reported the PTs to be at ΔE00 * (DE00) = 2.6–3.5 [18].

The acceptability threshold (AT) is a measure of the smallest color difference that can be considered acceptable by 50% of observers, while the other 50% would replace the restoration. Achieving a color match that falls at or below the 50:50 acceptability threshold is considered a desirable outcome in dental restorations, as it ensures that the restoration is acceptable and tolerable to most observers.

Rizzi suggests that below ΔE_94_ (Graphic Arts) = 0.67 the color difference is imperceptible, and that between 0.67 and 1 only an expert can distinguish differences. This threshold signifies that any color difference below this value in cases of sudden color changes is imperceptible to the human eye. In other words, when two samples with a uniform color surface are positioned next to each other but have different colors, a color difference of 0.67 ΔE_94_ in the dental color space is the minimum limit at which the human eye can detect the variation. However, if the samples are merely in close proximity but not physically touching, this threshold may increase up to three times. The same applies when there is a gradual and smooth transition between colors, as is often the case in dental restorations that use a bevel to seamlessly blend abrupt color transitions thanks to the translucency of thin composites.

According to Paravina et al. [19], the CIEDE2000 color-difference formula should be recommended for use in dentistry. Considering only the dental color space, Rizzi et al. have found that ΔE_94_ Graphic Arts is a color perceptual distance almost uniform along all axes in the dental color space [7]. This suggests the use of a general criterion of ΔE_94_ = 0.67 as color-discrimination value in the whole dental subspace, being the most isotropic formula of all inside this subspace [20]. For this reason, ΔE_94_ Graphic Arts formula has been applied in the present study.

It is important to underline that, despite the accuracy and ease of use of handheld color-matching instruments, the input of dental professionals remains crucial in this process. According to several authors, visual and instrumental techniques might be considered complementary tools, and their combination might help the clinician to reach predictable esthetic rehabilitations [21,22]. However, several factors in clinical settings, such as ambient illuminants, wrong handling, optical lens fogging, color background, and patient’s movement, etc., can still lead to imprecise color detection [23,24].

To improve the knowledge regarding visual and instrumental dental color-matching, the aim of the present investigation was to investigate the accuracy of a recently introduced colorimeter and to compare its ability in shade matching with human vision. The null hypothesis was that no difference exists among Optishade and the human vision. In addition, differences in color-matching among different operators were analysed.

## 2. Materials and Methods

### 2.1. Sample Preparation

**Figure 1:**
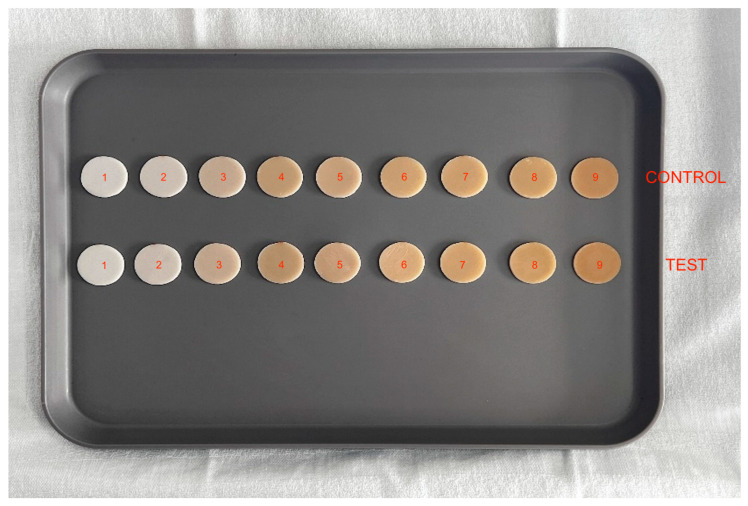
Nine control discs and nine test discs arranged following the Micerium chromatic scale (1-2-3-4-5-6-7-8-9) (incremental order).

The protocol of the study has been registered on the ISRCTN registry (ISRCTN85216751). A total of 18 disc-shaped composite samples (nine test and nine control) were produced (Figure 1).

The discs were created using a custom-designed transparent mold and an injection technique with flowable composite resin—Enamel plus Hri FlowHF (Micerium, Avegno, Genova, Italy), as reported in a previously published article [25]. The mold’s design enabled direct visual inspection during the injection process, preventing air bubbles and ensuring full space filling without any excess or unwanted thickness. The curing process took 1 min for both surfaces with a Elipar™ light-curing lamp (3M™, Maplewood, NJ, USA). Composites for both test and control groups came from the very same production batch, in order to prevent significant color variations resulting from the manufacturing process. A thin layer of light-curable BisCover™ LV varnish (Bisco Inc., Schaumburg, IL, USA) was applied to coat each disc, polymerized using the Elipar™ lamp as well.

The samples were assigned a numeric code according to the employed shade UD0 = disc 1, UD0, 5 = disc 2, UD1 = disc 3, UD2 = disc 4, UD3 = disc 5, UD3.5 = disc 6, UD4 = disc 7, UD5 = disc 8, UD6 = disc 9. The identifier was marked on the side with a light orange pen to minimize potential blending effects. The control discs were marked with numbers 1 to 9, while the test discs were indicated by symbols 1test to 9test. The symbols allowed for quick identification of the sample and its chief surface by the researchers throughout the entire testing phase.

All the discs had identical shape and size, measuring 30 mm in diameter (with an error of ±50 µm) and 2.384 mm in thickness (with an error of ±50 µm), to avoid color-contamination effects at the edges, such as the blending effect.

### 2.2. Optishade

Optishade Styleitaliano (Smile Line SA, St-Imier, Switzerland) is a recently introduced dental colorimeter that enables color evaluation of teeth, dental restorations, and materials used in dental procedures. The device consists of the main unit, in which are present the main components such as the lights, the sensor, and the camera button, the Capture Guide cone fitting the main unit, and, on top, a calibration cap snapped to the cone when the device needs to be calibrated.

Once the image is obtained through the device, a target square in the Optishade app (available on Apple Store version 4.11) can be moved with a finger to evaluate the CIELAB @D65 (or L*C*h* @D65, if preferred) values at any point on the tooth, with greater accuracy at the center of the crown. By storing all the CIELAB values of the most common composite, resin, and ceramic systems available on the market, the software immediately identifies the closest match, which is the material whose color is closest to that of the patient’s tooth.

Employing the “Compare” function offered within the Optishade app ΔE_94_ values were obtained and included in an error matrix (Table 1).

Within the error matrix, the related ΔE_94_ values coming from the test have been computed and clustered according to the amount of mismatch. The ΔE_94_ thresholds used to classify mismatches were chosen by the authors according to Manauta et al. [7], so that in the matrix the green color represents the match-cluster 0. That is a perfect match between the control and test discs (ΔE_94_ < 1), while all other colors indicate mismatches. Yellow indicates the match-cluster 1, which is mild mismatch (ΔE_94_ = 1 to 1.59); orange represents the match-cluster 2, which is moderate mismatch (ΔE_94_ = 1.6 to 2.69); red denotes the match-cluster 3, or severe mismatch (ΔE_94_ > 2.7); perceptibility and acceptability thresholds reported in literature [7] are taken into account.

The accuracy of Optishade to match test and control discs has been investigated by an expert operator (J.M.). L*a*b* values of the nine control discs have been stored in the Optishade app database, and an expert operator (J.M.) recorded the values for each of the test discs in a <10 lux dark room. After identification of the closest match for each of them by the Optishade app, data was recorded in an Excel sheet. This procedure was conducted six times with an incremental arrangement (1-2-3-4-5-6-7-8-9) and then with a randomly assorted arrangement (2-5-8-9-4-7-3-6-1) of the composite discs for each of the nine test discs, for a total of 108 matches.

### 2.3. Visual Accuracy Test

**Figure 2:**
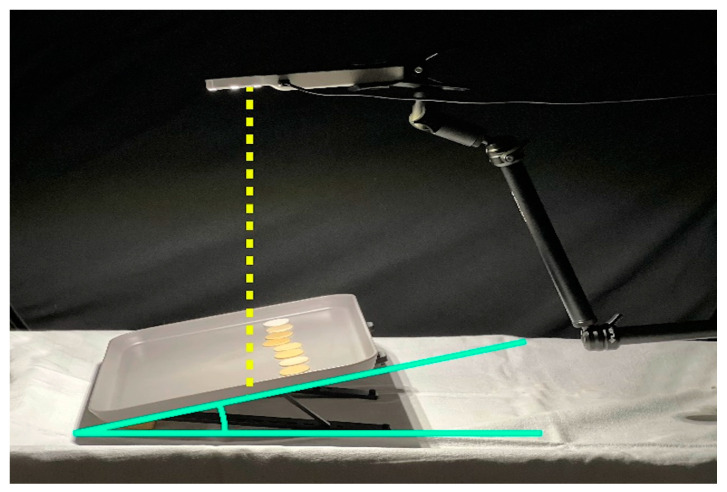
Study setup with the supporting table placed at a 30-degrees inclination for all subjects involved in the research.

To test visual accuracy in colour matching, the tests were conducted in a controlled environment at the Division of Prosthodontics and Implant Prosthodontics of the University of Genova, in complete darkness (<10 lx), with the sole illumination being provided by the selected illuminant. Although illuminance should be maintained within the range of 1000 to 2000 lx at the color-matching area, the present setting was chosen to facilitate the standardization of light intensity across the different hours of the day and along the different days during which the experimental phase took place. The chosen settings featured white walls and furnishings in neutral tones. The amounts of light present at the study setting were measured using the Lux Meter App version 2.1.1 (Apple store, Marina Polyanskaya) installed on an iPhone (Apple Inc., Cupertino, CA, USA). Data collection always took place under standard conditions with a distance of 30 cm between the illuminant and the tray supporting the samples (Figure 2).

The Smile Lite handheld light was chosen as the illuminant for the test, and it was secured with a mechanical arm above the tray to maintain a distance of 30 cm from the specimens. The Smile Lite emits a D55 light, with CRI = 89, whose intensity fluctuates depending on the residual battery charge. For this reason, and because of its tendency to shut down after prolonged use, it has been continuously connected to the power source using the dedicated charging cable.

The tray used to support the samples was colored in gray and exhibited a color variation on its surface of ΔE_94_ < 0.3. The tray’s gray color was essentially neutral under @D65, exhibiting extremely low +a* and +b* values. The tables where the observations were conducted were neutral in color.

The study was conducted by positioning the researcher in a composed seated position, orthogonally in front of the table, on which the tray containing the composite discs was placed. The surface of the discs with the most uniform color was placed facing upwards. The tray was tilted at a 30° angle with a specific support relative to the table and moved away to form a 30/0 degrees geometry with the viewing angle (Figure 2).

Italian dental students from fifth or sixth academic year, dentists, and dental technicians attending, working at, or collaborating with the Dental School of the University of Genova were asked to participate and were consecutively enrolled for the present research. The study involved individuals aged 18 to 70 years, from different branches and dental specializations. All participants were thoroughly informed about the study purpose and methodologies and signed an informed consent to participate in the test. Before starting, all participants underwent Ishihara tests to exclude from the study anyone showing color blindness, according to ISO/TR 28642:2016 standard [26]. No other exclusion criteria were applied.

Tester subjects were divided into PGS Group (Pre-Graduation Students), ECP Group (Early Career Professionals with less than 10 years of experience), and AP Group (Accomplished Professionals with 10 or more than 10 years of experience). They were seated one at a time, with the control discs positioned on the inclined tray, paying attention to keep the reference number unidentifiable. The control discs were lined up in the upper part of the tray by the investigator. Then, the investigator placed one by one the test discs at the center of the tray in front of the participant tester, who was asked to match them with the corresponding control disc (Figure 2). Following each match, the recently matched test disc was taken off the tray, and the next one was handed over. The visual inspection was conducted with the test and control discs placed apart, not in direct contact with each other. The test was executed first with the control discs arranged following the Micerium chromatic scale (1-2-3-4-5-6-7-8-9) (incremental order), and then the test was repeated placing the specimens at random with the same assortment for each tester (2-5-8-9-4-7-3-6-1) (assorted arrangement). The participants were forbidden to touch the control discs, and they had to bring the test disc close to the control disc they judged as color-matching.

### 2.4. Statistical Analysis

The analysis of human eye assessments involved recording the frequencies of accurate and inaccurate matches using the same scale arbitrarily chosen by the authors and described above (0: perfect match, 1 mild mismatch, 2 moderate mismatch, 3 severe mismatch). Each sample was compared to the other ones to assess any significant difference. Folded F-test was computed for capturing any difference in the number of matches of the sample distribution.

The percentage of matches achieved with the Optishade procedure were compared with those achieved by the human eye in both the incremental and the assorted arrangement. The Satterthwaite *t*-test was conducted.

## 3. Results

### 3.1. Outcomes of Optishade Accuracy Test

Optishade correctly matched all test and control discs. No mismatches were recorded through the device in this phase, neither in an incremental arrangement nor in an assorted arrangement.

### 3.2. Outcomes of Visual Accuracy Test

Seventy-two testers agreed to participate in the study and were enrolled. All of them were Italian. Two male testers presented weak positivity to the Ishihara test and therefore, they were excluded; therefore, 70 subjects performed the test. Mean age of participants was 37.4 ± 15.7 years (range 18–71 years); 44 participants were male (62.9%). Twenty-seven were in PGS Group (38.6%), 17 in the ECP Group (24.3%), and 26 in the AP Group (37.1%). The mean light intensity measured in the room was 397.5 ± 8.6.

Globally 1260 matches were analyzed; 984 (78%) were correct, 123 (10%) were mild mismatches, 67 (5%) were moderate mismatches, and 86 (7%) were severe mismatches. Table 2 reports the frequencies of correct matches and mismatches for each sample both in the incremental sequences and in the assorted one.

In the incremental order 77% were correct matches, 10% were mild mismatches, 6% were moderate mismatches, and 8% were severe mismatches. In the assorted order, 80% were correct matches, 9% were mild mismatches, 5% were moderate mismatches, and 6% were severe mismatches. Accuracy greatly varied depending on the shade examined. The easiest shades to identify were UD1 and UD6, achieving nearly maximum accuracy. On the contrary, UD2 and UD3.5 posed significant difficulties, recording a greater number of errors, including severe mismatches.

Results of the Folder test identified no differences in the comparison of frequencies among each sample, neither in the incremental order nor in the assorted one.

**Figure 3:**
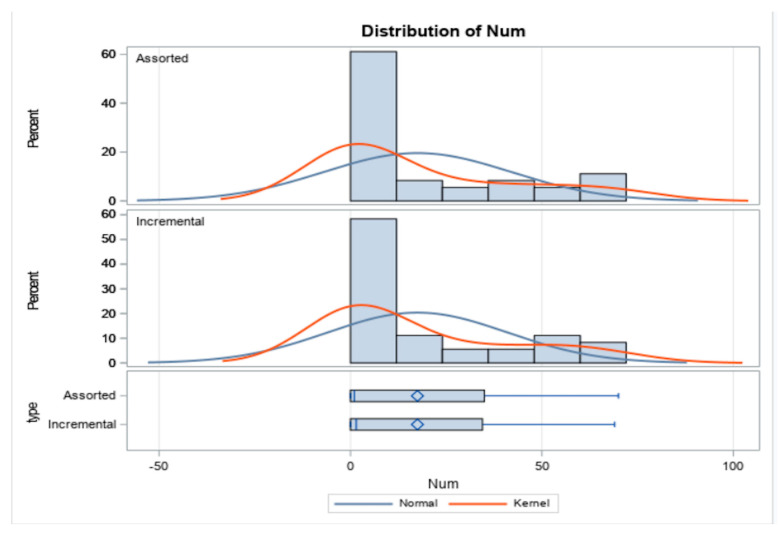
The chart shows the distribution of incremental and assorted number of matches. No significant difference between incremental and assorted number of matches were observed with a significant level of 5%.

Additionally, no significant difference between incremental and assorted number of matches were observed (Figure 3).

The effect of variables such as gender, age, and experience were analyzed. Data were summarized in Table 3.

Females were more likely to match the correct color than men (81% of females versus 76% of males), while, regarding age, individuals between age 51–65 registered the higher percentage of correct matches. Those under 30 years, and between 31–50, had a very high proportion of correct matches, while the individuals over 65 years matched the correct colors less frequently. Lastly, individuals of the PGS group were more likely to match the correct color than the ECP and AP groups. However, no statistically significant differences were identified among any of the considered groups (Table 4).

The Satterthwaite *t*-test reported a statistically significant difference with better results for Optishade both for the incremental (*p* = 0.0044) and the assorted arrangement (*p* = 0.0094).

## 4. Discussion

The present research investigated the accuracy of color-difference measurements of a new colorimeter (Optishade) and of human vision. The color difference between the samples in this study reflects the accuracy in measuring the color difference of identical samples. The null hypothesis must be rejected. In fact, Optishade performed successfully in all the matches, contrary to human eyes.

Multiple approaches were described to measure tooth color and, despite advances in technology, choosing the right color for human tooth aesthetic restoration remains a challenging task that is far from being fully automated [20,27,28]. The comparison of teeth with a shade guide is the most commonly employed method for evaluating tooth color [4], as it is a cost-effective and time-efficient process. The accuracy of visual tooth color measurement through shade matching can be substantially impacted by varying light sources, intensities, and metameric effects [29,30]. Other subjective factors such as color blindness, age, weariness, nutrition, emotions, medications, and binocular disparity are of concern as well [31]. Even the setting whereby the tooth is observed is critical since the color of the gums and lips [21], and even the tongue position [32], can alter its interpretation. In this context, the market started to design new tooth color detection devices with the aim of overcoming the visual shade-matching limitations. Nowadays, most popular objective shade-matching techniques includes spectrophotometers [33,34,35,36], colorimeters, post-production photographic software, and, more recently, intraoral digital scanners. Regardless of the device, the basis for all instrumental color-measurement techniques is the hypothetical standard observer.

The importance of the operator experience is debated. Research showed that it not necessarily leads to superior color-matching skills in dentistry, although numerous authors claim the contrary [37,38]. In addition, the age and health of the eyes can also play a significant role in color perception, with younger individuals typically having better color vision than older individuals [39]. In fact, aging, cataract, and pharmacological treatments can commonly lead to an acquired xanthochromic view condition [40]. Other acquired color-vision deficiencies can follow neurologic and systemic diseases, such as multiple sclerosis [41] or type II diabetes [42]. In addition, it is worth noting that most authors suggest that there is no scientific evidence to support the idea that females have superior color-matching ability compared to males among trichromats individuals [43,44], even though others claims that this difference exists [45]. However, it is more common for males to be affected by color deficiency, with a prevalence of 1 in 12 (8%) compared to females with a prevalence of 1 in 200 (0.5%) [46].

Dealing with Optishade’s accuracy, the device found the lowest ΔE_94_ color difference among the control discs in the database and successfully identified the correct match for test discs in 100% of cases, both in the incremental stage and in the assorted stage. This is obvious because the colorimeter can check one color at a time and, therefore, each disc is out of context in both the incremental and assorted stages. The colorimeter might be a useful and effective device for clinicians, overcoming possible limitations of visual matching due to color vision deficiencies, eye fatigue, age, viewing conditions, length of exposure, or afterimage effects.

In fact, when visual determination was investigated, only 78% of the correct responses were obtained. The 10% of mismatches belonged to the match-cluster 1 (ΔE_94_ = 1 to 1.59), indicating mild mismatches. However, the 8% belonging to match-cluster 2 (ΔE_94_ = 1.6 to 2.69) indicate a significantly higher severity of errors, exceeding the perceptibility threshold [19,20]. This type of mismatch should be easily recognizable by an experienced eye; however, the specific experimental setting might have affected the different outcomes in different studies. Lastly, the remaining 4% of errors were of a severe mismatch (ΔE_94_ > 2.7), going beyond the acceptability threshold. This data agrees with scientific literature, since accuracy of shade matching by observers has been estimated to be between 58% and 86% [47,48] but still inferior than those of dental spectrophotometers [49].

One of the most difficult samples to match was 4test (UD 2), with an error rate of 47% during Stage 1B (incremental sequence) and 31% during Stage 2B (assorted sequence). Regarding the errors, it was consistently associated with disc 5control and never with 3control. This is to be expected, considering that the color difference with 5control samples is close to the threshold of perceptibility (ΔE_94_ = 1.2), whereas it is significantly higher compared to 3control (ΔE_94_ = 5.99). There was only one instance where it was associated with 6control (ΔE_94_ = 1.9) during Stage 2B.

Interestingly, 5test (UD 3) was correctly associated in only 76% (Stage 1B) and 79% (Stage 2B) of instances. This took place even though the ΔE_94_ = 1.17 between 5test and 4control is entirely overlapping with that between 4test and 5control.

Also, the matching of 6test (UD 3.5) proved to be a challenging task, as it was incorrectly matched in over half of the cases (54%). It should be noted that the ΔE_94_ between disc 6control and 6test was the highest when compared to the rest of the discs. It is plausible that there was a manufacturing defect in the composite material used. This is suggested by the authors, as evidenced by the error matrix, where it can be observed that the color difference between 7control and 6test (ΔE_94_ = 1.27) is much smaller compared to the difference between 6control and 7test (ΔE_94_ = 2.3). Similarly, the ΔE_94_ between 6control and 5test (ΔE_94_ = 2.19) is significantly greater than that between 5control and 6test (ΔE_94_ = 3.3). This could suggest that a shift in L*a*b* values towards those of the seven samples has occurred, which is confirmed observing a general decrease in L* values and an increase in b* values as they approach to the L*a*b* values of the seven samples. For this reason, in the central portion of the matrix, there is no mirror symmetry, explaining, for example, why 6test has been more frequently associated with 7control than the association between 7test and 6control.

Concerning the sample 1test (UD 0), which displays distinct characteristics such as high L* values (around 81) and extremely low a* (around 0) and b* values (around 3.5), it was commonly paired with 2control (20%), resulting in the formation of match-cluster 3 (ΔE_94_ = 4.06), but solely during Stage 1B. In fact, during Stage 2B, it was incorrectly matched in only 4% of cases. The same pattern was observed for 2test, which was incorrectly associated with 1control in 11% of matches during Stage 1B and, remarkably, had 0% errors during Stage 2B. The incremental position seems to significantly affect the perception of gray values, as well-known as the Chevreul Illusion. Additionally, it is intriguing to observe that this type of error was committed, except for one instance, only by male testers, but this probably occurred by chance.

Sample 8test (UD 5) achieved only 74% correct matching during Stage 1B, but unlike the other shades, this percentage dropped to 59% during Stage 2B. These data are highly intriguing as the color differences with 7control (ΔE_94_ = 3.17) and 6control (ΔE_94_ = 4.13), the only two samples it was incorrectly matched with, are much larger compared to those between samples 5 and 4, which yielded similar, if not better, results. Furthermore, it is noteworthy that 8test was never matched with 9control, despite belonging to the match-cluster 2 (ΔE_94_ = 2.09). The color of the discs is given by the base color of the composite and the surface reflection due to its surface morphology, like scratches or minor irregularities if the surface was not polished thoroughly. Darker composites tend to reflect less base color light emphasizing these surface reflections. This effect is less noticeable with Specular Component Excluded, like the instrumental measurements conducted in this experiment, where the base color was dominant. However, human observers might be fooled by the surface reflections, and this could explain why a darker shade is confused for a lighter one.

Despite having a color difference that falls within a match-cluster 2 with its closest sample, 8control, the 9test disc (UD 6) was incorrectly matched only two times in Stage 1B and four times in Stage 2B. Out of these six errors, two were made by associating it with 7control (ΔE_94_ = 4.59). The 9control placement at the far end of the tray in incremental sequence, as well as its proximity to sample 8control and 4control in assorted sequence, likely made 9test easier to recognize compared to other samples.

Lastly, 3test was the sample that raised the least doubts. Only two errors were made, likely due to inattention, by two different testers. The ΔE_94_ measurements between it and the most similar samples (2control and 4control) are well above the threshold of acceptability, leading to its correct identification (ΔE_94_ > 5.89).

Considering these findings, it becomes evident that on the whole, darker hues have been more susceptible to errors than lighter ones. Despite seemingly contradictory to the outcomes by Milagres [50], it is crucial to bear in mind that the ΔE_94_ between UD1, UD2, UD3, and UD4 were notably higher than those between UD6, UD7, UD8, and UD9 concerning the Micerium composite line.

Overall, the evaluation in Stage 1B proved to be slightly more challenging (77% correct matches) compared to Stage 2B (80% correct matches), with a higher frequency of severe errors by more than two percentage points (8%).

The extensive array of errors shows how the subjective nature of visual inspection can mislead clinicians, even when working in a simplified scenario such as the one described in the present study. The test discs, in fact, had been manufactured with surface uniformity and dimensions that aimed to prevent any interference with the background and the surrounding environment, making them as neutral as possible. Into the oral setting, several confounding factors are present, including the color of the gums and lips [21], and even the tongue position [32] can alter dental color interpretation. This couldn’t happen in this study. Nevertheless, there were a significant number of errors belonging to match-cluster 2 that should have been easily noticeable, as well as those belonging to match-cluster 3 that did not meet the standards of aesthetic acceptability.

Disparities in the skill to accurately match colors were not identified depending on the gender of the test participants. Even if females displayed a slightly higher proficiency (81% correct matches) compared to males (76% correct matches), no statistically significant differences were identified. Some researchers report gender as a critical factor and assert the superiority of females in color perception [45,50,51]. However, results of the present research reveal that gender and age have no significant effects on color discrimination. Our results are in accordance with Reyes et al. [44], Liu et al. [52], and Alomari et al. [43].

The aging process causes the cornea and lens of the eye to develop a yellowish hue, leading to a biased yellow-brown perception, which negatively affects human ability to match shades. The transition begins at around 30 years of age, becomes increasingly evident at 50, and assumes medical significance once individuals enter their 60s. After turning 60, a considerable proportion of people struggle with discerning blue and purple colors. The aging process causes images to appear more yellowish and brownish, leading to difficulties in color perception [39]. However no statistically significant differences were reported in the present study.

The clinician’s experience probably has a strong effect on the visual method’s accuracy and precision of the aforementioned criteria [37,53], while variables of shade guide and the lighting condition become more significant for amateurs. This brought several authors to the conclusion that color education, often ignored in dental education, is strongly recommended to be deepened [54,55,56]. However, our results did not find any statistically significant differences among the groups.

When considering professional branch, important distinctions were observed among the practitioners. The dental technicians (*n* = 6) achieved the highest percentage of cluster 0 matches (80%). Among dentists, specialists in conservative dentistry achieved a cluster match of 0 in 78% of cases, followed by orthodontists (*n* = 4) with 76%, implant prosthodontists (*n* = 9) with 75%, and general dentists (*n* = 1) ranking towards the lower end. The sample size of the testers is inadequate to establish the significance of these findings. For some reason, students (*n* = 8) performed better than professionals (80%).

Considering possible limitations of the present research, it is worth mentioning the concerns regarding the artisanal production of sample 6test, which displayed slight deviations from the ideal L*a*b* values. This difference, although imperceptible to the human eye, may have made it more challenging to properly match it with sample 6control and might have influenced the obtained values. Also, the random arrangement of Stage 2B plays a role, and changing the sample order may influence the test result due to color neighbor interplay.

In addition, the scale of measurement used (indicating 0 a perfect match, 1 mild mismatch, 2 moderate mismatch, and 3 severe mismatch) is not based on a standard agreement scale, which typically utilizes percentages. This could have influenced the interpretation of results, particularly regarding severe mismatches in visual shade matching.

Finally, it must be underlined that the uniqueness of the study setting, although designed in accordance with literature suggestions and ISO regulations, is not comparable to a clinical setting. This implies that the quantity and severity of errors committed could vary when it comes to patients’ teeth, either improving or worsening the outcomes. Moreover, the D55 light, although recommended for research of this nature, produced reflections on samples that, in some cases, disrupted the tester’s vision, exacerbated by the photopolymerizable paint layer, necessitating a modification of the viewing distance. This could have had additional implications on the performance of the tester subjects, without affecting Optishade due to the presence of the polarizing filter. Additionally, a power analysis to determine the sample size was not conducted.

## 5. Conclusions

In conclusion:

- The instrumental shade matching evaluated proved to be significantly more reliable than the human visual system.
- Subjectivity of human color vision has led to a level of accuracy that falls short of optimal and was significantly lower compared to the instrumental evaluation.
- A higher occurrence of mismatches was noted for intermediate composite shades without a statistically significant difference.
- No statistically significant differences were reported for age, sex, and experience.
- Further research is needed to determine whether the same outcomes are achieved in a clinical setting directly on patients.

## Figures and Tables

**Table 1:** Error matrix representing ΔE_94_ test and control discs.

	1control	2control	3control	4control	5control	6control	7control	8control	9control
1 test	0.1	4.06	10.8	16.89	16.67	18.59	20.64	23.44	25.58
2 test	4.02	0.23	6.91	13.01	12.71	14.66	16.64	19.34	21.35
3 test	10.7	6.53	0.22	6.22	5.83	7.74	9.58	12.22	14.02
4 test	16.87	12.65	5.89	0.27	1.17	1.9	3.49	6.24	8.1
5 test	16.89	12.62	5.76	1.2	0.14	2.19	3.61	6.25	7.94
6 test	19.82	15.51	8.64	2.69	3.3	0.57	1.27	4.13	5.89
7 test	20.87	16.5	9.51	3.54	3.88	2.3	0.13	2.87	4.59
8 test	23.66	19.21	12.1	6.26	6.51	5.34	3.17	0.25	2.09
9 test	25.66	21.06	13.8	7.97	8.07	6.77	4.69	2.17	0.14

**Table 2:** Matching accuracy carried out by testers regarding both the incremental and assorted sequences (0: perfect match, 1 mild mismatch, 2 moderate mismatch, 3 severe mismatch).

		Incremental Order		Assorted Order	
Sample	Match-Cluster	*n*	%	*n*	%
1 (UD 0)	0	56	80%	67	96%
	1	0	0%	0	0%
	2	0	0%	0	0%
	3	14	20%	3	4%
2 (UD 0.5)	0	62	89%	70	100%
	1	0	0%	0	0%
	2	0	0%	0	0%
	3	8	11%	0	0%
3 (UD 1)	0	69	99%	69	99%
	1	0	0%	0	0%
	2	0	0%	0	0%
	3	1	1%	1	1%
4 (UD2)	0	37	53%	48	69%
	1	0	0%	21	30%
	2	29	41%	1	1%
	3	4	6%	0	0%
5 (UD 3)	0	53	76%	55	79%
	1	16	23%	14	20%
	2	1	1%	0	0%
	3	0	0%	1	1%
6 (UD 3,5)	0	32	46%	44	63%
	1	0	0%	0	0%
	2	37	53%	25	36%
	3	1	1%	1	1%
7 (UD 4)	0	52	74%	42	60%
	1	16	23%	23	33%
	2	0	0%	0	0%
	3	2	3%	5	7%
8 (UD 5)	0	52	74%	41	59%
	1	18	26%	0	0%
	2	0	0%	0	0%
	3	0	0%	29	42%
9 (UD 6)	0	68	97%	66	95%
	1	0	0%	0	0%
	2	1	1%	3	4%
	3	1	1%	1	1%

**Table 3:** Matching accuracy depending on sex, age, and work experience. (0: perfect match, 1 mild mismatch, 2 moderate mismatch, 3 severe mismatch). PGS Group (Pre-Graduation Students), ECP Group (Early Career Professionals with less than 10 years of experience), and AP Group (Accomplished Professionals with 10 or more than 10 years of experience).

Variable	Group	Match-Cluster	% of Individuals
Sex	Female (n. 25)	0	81%
		1	9%
		2	8%
		3	2%
	Male (n. 45)	0	76%
		1	10%
		2	8%
		3	5%
Age	≤30 years (n. 43)	0	79%
		1	8%
		2	9%
		3	4%
	31–50 years (n. 12)	0	77%
		1	10%
		2	7%
		3	7%
	51–65 years (n. 11)	0	80%
		1	9%
		2	9%
		3	2%
	>65 years (n. 4)	0	67%
		1	19%
		2	7%
		3	7%
Experience	PGS Group (n. 31)	0	80%
		1	8%
		2	8%
		3	4%
	ECP Group (n. 22)	0	78%
		1	9%
		2	8%
		3	5%
	AP Group (n. 17)	0	76%
		1	12%
		2	8%
		3	4%

**Table 4:** Results of the Folded F-test for matching accuracy depending on sex, age, and work experience.

Variable	Group 1	Group 2	*p*-Value Folded F
Sex	Female	Male	0.8856
Age	<30 years	31–50 years	0.9446
Age	<30 years	51–65 years	0.9738
Age	<30 years	>65 years	0.7110
Age	31–50 years	51–65 years	0.9185
Age	31–50 years	>65 years	0.7629
Age	51–65 years	>65 years	0.6870
Experience	PGS Group	ECP Group	0.9527
Experience	PGS Group	AP Group	0.9083
Experience	ECP Group	AP Group	0.9554

## Data Availability

Data available on request.
